# Effects of slope on lower-limb bilateral asymmetry and dynamic stability during running

**DOI:** 10.3389/fphys.2026.1947077

**Published:** 2026-09-08

**Authors:** Lu Zhang, Zhengxiong Zhu, Ming Li, Hongshen Wang

**Affiliations:** 1 School of History and Culture, Chengdu Sport University, Chengdu, Sichuan, China; 2 School of Physical Education and Health, Lijiang Normal College, Lijiang, Yunnan, China; 3 College of Education and Sports Sciences, Yangtze University, Jingzhou, Hubei, China

**Keywords:** injury risk, joint moment, margin of stability, musculoskeletal modeling, tissue load

## Abstract

**Purpose:**

To examine differences in lower-limb bilateral asymmetry and dynamic stability across running slopes at a fixed speed.

**Methods:**

A repeated-measures secondary analysis of a publicly available multi-slope running biomechanics dataset was performed. Nineteen healthy runners ran at 2.78 m·s⁻¹ on five slopes from −6° to +6°. Asymmetry in five peak loading variables was determined using the percentage difference method. For the margin of stability (MoS) analysis, anteroposterior pelvic velocity was expressed relative to the treadmill belt by adding the belt speed of 2.78 m·s⁻¹; no belt-speed correction was required for the mediolateral calculation. Slope effects were analyzed using linear mixed-effects models with Benjamini–Hochberg control of the false discovery rate.

**Results:**

Significant slope effects were found for the asymmetry of Achilles tendon strain, knee extension moment, and patellofemoral stress and for both anteroposterior and mediolateral MoS (q < 0.05), but not for vertical ground reaction force or ankle plantarflexion moment asymmetry. Anteroposterior MoS increased monotonically, becoming less negative from −0.573 ± 0.021 m (−6°) to −0.523 ± 0.033 m (+6°; F_4_, _72_ = 77.74, q < 0.001, partial η² = 0.81). Mediolateral MoS decreased from 0.062 ± 0.022 m (−6°) to 0.052 ± 0.031 m (+6°; F_4_, _72_ = 3.50, q = 0.016, partial η² = 0.16). Achilles tendon strain asymmetry decreased from downhill to uphill running, whereas knee extension moment and patellofemoral stress asymmetry were greatest near mild uphill running.

**Conclusions:**

Lower-limb bilateral load distribution and direction-specific mechanical margins varied across slopes. Load magnitude and bilateral asymmetry did not change in parallel, indicating that asymmetry may provide information complementary to absolute load. The strongly negative anteroposterior MoS values are mechanically plausible during running and should not, by themselves, be interpreted as evidence of global instability.

## 1 Introduction

Running is one of the most popular and accessible forms of physical activity worldwide (Kakouris et al., 2021). Among its forms, trail running, which is performed on non-level terrain such as slopes and mountainous ground, has seen a marked increase in participation over the past decade. This growth has been accompanied by a considerable burden of lower-limb injury, with injuries concentrated at the knee, ankle, and other lower-limb sites (Viljoen et al., 2022). Slope systematically alters the distribution of load within the lower limb. During uphill running, the lower limb must perform more positive work to raise the body, and loading of the ankle and Achilles tendon increases (Vernillo et al., 2017). Downhill running, in contrast, is dominated by energy dissipation, with the knee extensors bearing a greater eccentric load that has been associated with the risk of overuse injury (DeVita et al., 2008; Vernillo et al., 2017). Slope therefore does not simply alter running intensity; it changes the way load is distributed among the structures of the lower limb.

Over repeated running exposures, this redistribution may have unequal consequences for the two limbs. If one limb consistently bears a greater share of joint or tendon loading, it may accumulate greater structure-specific mechanical exposure (Van Hooren et al., 2024), potentially contributing to unilateral overuse when cumulative load exceeds tissue capacity (Bertelsen et al., 2017). Such bilateral asymmetry may reflect individualized neuromuscular control and compensatory load-sharing strategies rather than fixed structural differences alone (Zhang et al., 2025). It is also commonly considered when assessing lower-limb function and athletic performance (D’Hondt et al., 2024, 2026b). However, studies of bilateral asymmetry during running have largely been limited to level running or different running speeds. For example, Mo et al. (2020) reported that running asymmetry varied with speed and showed marked between-individual differences. Conversely, research on slope running has primarily examined slope-related changes in overall joint mechanics and external loading, rather than how these loads are distributed between the left and right limbs. Park et al. (2019), for instance, found that downhill running increased knee flexion while reducing ankle plantarflexion and propulsive ground reaction force compared with level running. This limitation is particularly important because bilateral asymmetry is highly task-specific: both its magnitude and direction may vary across movement tasks and outcome measures (Chapelle et al., 2022; D’Hondt et al., 2025a). Consequently, asymmetry observed during level running may not generalize to uphill or downhill running. Whether the magnitude and direction of bilateral asymmetry in lower-limb mechanical loading vary across slopes therefore remains to be established.

In addition to altering lower-limb loading, slope has also been shown to influence dynamic stability during running. Using the same publicly available dataset, Promsri (2025) found that treadmill gradient affected local dynamic stability during mid-stance, with decline running exhibiting lower stability than incline running. Dynamic stability can be viewed as the mechanical outcome of balance control, which comprises the active neuromuscular processes used to maintain or restore locomotor equilibrium. The functional importance of these processes is evident in both perturbation recovery and energy expenditure. Experimental and simulation evidence indicates that trunk-control mechanisms contribute to accommodating sudden changes in surface height and restoring locomotor equilibrium following step-down perturbations (Drama et al., 2020). These balance-control processes also impose measurable energetic demands: external lateral stabilization reduced step-width variability and the net metabolic cost of running, demonstrating that the active maintenance of lateral balance contributes to running energy expenditure (Arellano and Kram, 2012). One widely used approach to evaluating dynamic stability is the extrapolated center of mass (XCoM) and margin of stability (MoS) framework proposed by Hof et al. (2005), which describes the position of the XCoM relative to the boundary of the base of support in the anteroposterior (AP) and mediolateral (ML) directions. Nevertheless, the available evidence remains limited. Promsri (2025) evaluated local dynamic stability using the largest Lyapunov exponent of movement components but did not quantify the spatial stability margin between the XCoM and the base of support. Other studies examining slope-related balance-control mechanics have focused primarily on walking; for example, Silverman et al. (2012) reported slope-dependent changes in whole-body angular momentum during incline and decline walking. Therefore, how the AP and ML margins of stability vary across running slopes remains unclear.

Given the functional relevance of inter-limb load distribution and dynamic stability and the limited evidence regarding their responses to slope, the present study conducted a secondary analysis of a publicly available running biomechanics dataset in which participants ran at a fixed speed across five slopes (−6°, −3°, 0°, +3°, and +6°). Bilateral asymmetry was quantified for five peak stance-phase variables spanning external, joint-level, and tissue-level loading: vertical ground reaction force (vGRF), ankle plantarflexion moment, Achilles tendon strain, knee extension moment, and patellofemoral stress. Dynamic stability was quantified as the AP and ML margins of stability. Specifically, the study aimed to: (1) determine whether the magnitude of lower-limb bilateral asymmetry differs across slopes in a site-specific manner; (2) examine whether the direction of asymmetry is consistent across participants; and (3) determine whether the AP and ML margins of stability differ across slopes. We hypothesized, first, that bilateral asymmetry would differ across slopes in a site-specific manner, such that significant differences would be observed for some, but not all, joint- and tendon-related loading variables; and second, that dynamic stability during running would differ significantly across slopes, most notably in the AP margin of stability.

## 2 Methods

### 2.1 Data source and experimental design

This study was a repeated-measures secondary analysis of publicly available data. The data were obtained from an openly accessible running biomechanics database described in a peer-reviewed publication and deposited in the Open Science Framework (Van Hooren and Meijer, 2024; Van Hooren et al., 2023; project DOI: 10.17605/OSF.IO/7QBXC; https://osf.io/7qbxc/). The database comprises three-dimensional kinematics, ground reaction forces, surface electromyography, and musculoskeletal modeling outputs collected from healthy runners across different running speeds, surface slopes, cadences, and forward trunk lean conditions. The present analysis included only the slope experiment, with running speed fixed at 2.78 m·s⁻¹. Five slope conditions were compared: −6°, −3°, 0°, +3°, and +6°. All other speed, cadence, and forward trunk lean conditions were excluded, making slope the only running-condition factor examined in the present study.

### 2.2 Participants

The original sample comprised 19 healthy participants (10 men and 9 women; age: 23.6 ± 3.7 years; height: 174.9 ± 9.2 cm; body mass: 67.2 ± 10.4 kg), whose performance levels ranged from untrained to Olympic level (Van Hooren and Meijer, 2024). All participants completed the five slope conditions described above, and their data were included in all outcome analyses. The original data collection was approved by the local ethics committee at Maastricht University (approval No. 2019-1138) and conducted in accordance with the Declaration of Helsinki. All participants provided written informed consent before data collection (Van Hooren and Meijer, 2024). The present study involved only a secondary analysis of previously published, de-identified data; no new recruitment or data collection was undertaken, and no personally identifiable information was accessed.

The sample size was determined by the original public dataset rather than by an *a priori* power analysis. To evaluate the effect size detectable with the available sample, a sensitivity analysis was conducted in G*Power 3.1.9.7 (F test, repeated-measures analysis of variance, within-participant factor). With α = 0.05, statistical power of 0.80, five slope levels, and a correlation among repeated measures set to 0.50, the smallest detectable effect size with 19 participants was Cohen’s f = 0.259 (partial η² ≈ 0.06).

### 2.3 Original data collection

The original data were collected by the authors of the database in a Computer Assisted Rehabilitation Environment (Motek, The Netherlands), which incorporates a dual-belt instrumented treadmill (ForceLink, The Netherlands; ground reaction forces sampled at 1000 Hz) and a 12-camera three-dimensional motion capture system (Vicon Nexus 2.1, Oxford Metrics Group, UK; sampled at 100 Hz) (Van Hooren and Meijer, 2024). Participants wore their own running shoes, and 26 reflective markers of 14 mm diameter were attached to the lower limbs and trunk, with marker placement based on a modified Human Body Model v2 (van den Bogert et al., 2013; Van Hooren and Meijer, 2024). After an 8 min familiarization period on the treadmill at 2.78 m·s⁻¹, participants completed the speed and slope conditions in randomized order, each lasting 1 min. All five slope trials used in the present study were recorded at 2.78 m·s⁻¹, with the level (0°) condition corresponding to the 2.78 m·s⁻¹ level running trial in the database (Van Hooren and Meijer, 2024).

### 2.4 Data processing

Marker labeling was performed by the authors of the database in Vicon Nexus, gaps in marker trajectories were filled using a combination of pattern fill, spline fill, and rigid body fill, and the trajectories were smoothed with a Woltring quintic spline (Woltring, 1986; Van Hooren and Meijer, 2024). The processed marker and external force data were imported into OpenSim 3.3 (Delp et al., 2007) and analyzed using a modified Catelli full-body musculoskeletal model (Catelli et al., 2019), which comprises 22 rigid segments, 37 degrees of freedom, and 80 muscles, with muscle moment arms modified for tasks involving large hip and knee flexion. Joint angles were obtained by inverse kinematics and net joint moments by inverse dynamics, whereas muscle forces were computed by dynamic optimization accounting for muscle activation, force–length–velocity relationships, and tendon compliance (De Groote et al., 2016). Joint contact forces were derived by combining the intersegmental forces from inverse dynamics with the muscle forces from dynamic optimization (Steele et al., 2012; Van Hooren and Meijer, 2024). Patellofemoral stress and Achilles tendon strain were computed by the authors of the database using their published tissue load models, described in detail in the original tissue load paper (Van Hooren et al., 2024).

For the MoS analysis, the three-dimensional trajectories of the pelvic and foot markers were obtained from the corresponding.trc files in the “03. GRF, markers, event files” directory. The marker trajectories had already been gap-filled and smoothed by the database authors using a Woltring quintic spline, and no additional filtering was applied before numerical differentiation in the present analysis. The raw C3D files were not used in the present analysis. The per-trial workbooks in the non-normalized directory were used only for the tissue-loading outcomes, with Achilles tendon strain and patellofemoral stress obtained from the worksheet labeled “FS.” To verify the consistency of the tissue-loading data source, per-stance peak Achilles tendon strain and patellofemoral stress re-extracted from these workbooks for Sub_01 to Sub_16 were compared value by value with the corresponding results from the separate tissue-load files in the database; the maximum absolute difference was less than 6 × 10⁻¹^4^ in all cases. All outcomes were computed within each actual stance phase and subsequently aggregated to the participant level.

### 2.5 Stance identification and quality control

Instants of initial contact and toe-off for the left and right feet were taken directly from the event files provided by the original database rather than being re-detected in the present study using a ground reaction force threshold, and each stance phase was defined by a paired initial contact and toe-off. Quality control was applied in sequence. First, events were excluded if initial contact or toe-off was missing. If toe-off did not follow initial contact or if stance time fell outside the range of 0.10 to 0.50 s, the corresponding events were also excluded. Second, only stance phases falling entirely within the time window over which the corresponding ground reaction force, inverse dynamics, and marker trajectory data were jointly valid were retained. No fixed-duration segment of each one-minute trial was selected. Instead, all complete stance phases within the time interval jointly covered by the relevant data streams were included in the analysis. Finally, a plausible range of 0.50 to 5.00 times body weight was applied to the peak vGRF of each stance phase as a screening criterion.

After quality control, 13, 152 stance phases were included for the calculation of vGRF, joint moments, and dynamic stability. Because each FS worksheet provides only 50 s of continuous tissue load output, tissue load peaks were computed only for stance phases that also fell within this valid time window; 12, 792 stance phases met this criterion, and the remaining 360 fell outside the window and did not contribute to the tissue load calculations. At least 41 tissue load stance phases were retained for every participant × slope × limb cell, so that all 95 participant × slope cells from the 19 participants were available for the analysis of Achilles tendon strain and patellofemoral stress. No imputation was performed for data outside the valid time window, and all included stance phases retained their original initial contact, toe-off, and actual stance time.

On this basis, five peak outcomes were extracted from each eligible stance phase. Peak vGRF was the maximum of the vertical ground reaction force for the corresponding limb, normalized to body mass and gravitational acceleration (9.80665 m·s⁻²) and expressed in multiples of body weight. Peak ankle plantarflexion moment and peak knee extension moment were the maxima, within the stance phase, of the absolute sagittal-plane net moment at the ankle and knee, respectively, normalized to body mass and expressed in N·m·kg⁻¹. Peak Achilles tendon strain and peak patellofemoral stress were the within-stance maxima of AchillesTendon_Strain and Patellofemoral_Stress for the corresponding limb in the FS worksheet, expressed in % and MPa, respectively. For each participant, slope, and limb, the arithmetic mean of the peaks across all eligible stance phases within the valid time window of the relevant data stream was first computed to yield a right-limb mean (R) and a left-limb mean (L). The experimental unit for statistical analysis was therefore the participant rather than the individual stance phase. Previous studies have reported good within- and between-day repeatability for peak force measures across treadmill walking and running speeds (intraclass correlation coefficients [ICCs] > 0.878), with smallest detectable changes below 135 N during running (Nüesch et al., 2018). For lower-limb joint moments during running, Alenezi et al. (2016) reported within-day ICCs of 0.64–0.89, between-day ICCs of 0.58–0.91, and standard error of measurement (SEM) values of 0.07–0.39 N·m·kg⁻¹; running ground reaction force measures showed ICCs ≥ 0.84. Methodological reviews further emphasize the importance of considering measurement reliability when interpreting bilateral asymmetry (Bishop et al., 2023; Afonso et al., 2025).

### 2.6 Bilateral asymmetry and directionality

Bilateral asymmetry magnitude was quantified using the standard percentage difference method recommended for separately obtained unilateral outcomes (Bishop et al., 2018):


$$\text{Asymmetry Index }(\%)\;=\;100\times \frac{\text{Higher}-\text{Lower}}{\text{Higher}}\;$$

where Higher value and Lower value represent, respectively, the larger and smaller of the two limb-specific mean peak values for each participant, outcome, and slope. The resulting percentage represents the magnitude of asymmetry independently of its direction. For the separate analysis of asymmetry direction, the right-limb (R) and left-limb (L) values were retained according to anatomical side. Direction was examined descriptively by reporting the raw peak values for R and L and the proportion of participants with R > L at each slope. Because the side producing the higher value varied across participants and outcomes, direction was interpreted separately for each outcome. No inferential comparisons between limb sides were performed at individual slopes.

### 2.7 Dynamic stability

Dynamic stability was assessed using the XCoM–MoS framework proposed by Hof et al. (2005). The geometric center of four pelvic markers—the left and right anterior and posterior superior iliac-spine markers—was used as a proxy for whole-body center-of-mass position. The common laboratory coordinate system was defined with X mediolateral, Y anteroposterior, and Z vertical, with running proceeding in the negative-Y direction. Pelvic velocity was obtained by numerical central differentiation of the 100-Hz trajectories, with forward and backward differences used at the endpoints. The XCoM was computed as:


$$\text{XCoM}=\text{p}+\frac{\text{v}}{{\text{ω}}_{0}},{\text{ ω}}_{0}=\sqrt{\frac{\text{g}}{\text{h}}}$$

where p is the pelvic-proxy position, v is its velocity relative to the support surface, g = 9.80665 m·s⁻², and h is the pelvic-proxy height above the inclined treadmill surface. Because running proceeded in the negative-Y direction, forward position was defined as negative Y, and forward velocity was obtained by differentiating this position with respect to time. For the AP calculation, the constant treadmill-belt speed of 2.78 m·s⁻¹ was added to the laboratory-frame forward pelvic velocity to express velocity relative to the moving support surface (Curtze et al., 2024). No belt-speed correction was required in the ML direction. For each slope, pelvic-proxy height was calculated as the vertical distance between the pelvic center and the inclined treadmill plane at the same AP position. AP MoS was defined as the signed distance at initial contact between the stance-foot second-metatarsal marker and the AP XCoM. ML MoS was defined as the minimum signed distance during stance between the stance-foot fifth-metatarsal marker and the ML XCoM. Values were averaged across all valid left and right stance phases for each participant and slope. Negative AP MoS indicates that the XCoM lies anterior to the forefoot boundary at initial contact; such values are mechanically plausible during running and are not, by themselves, evidence of global instability (Curtze et al., 2024).

### 2.8 Statistical analysis

Statistical analyses were performed in R version 4.3.3, and data are presented as mean ± standard deviation. The primary analysis examined differences in the asymmetry index across slopes. Separate linear mixed-effects models were fitted for the five primary asymmetry outcomes and the two dynamic stability outcomes using the lme4 package (Bates et al., 2015). In each model, the corresponding outcome was specified as the dependent variable, slope (−6°, −3°, 0°, +3°, and +6°) as a categorical fixed effect, and participant as a random intercept. Omnibus tests of the slope effect were conducted using the Satterthwaite approximation for degrees of freedom implemented in the lmerTest package (Kuznetsova et al., 2017). The results are reported as F values, numerator and denominator degrees of freedom, and partial η². To control the risk of false-positive findings arising from multiple primary outcomes, the seven omnibus tests of slope—comprising five asymmetry outcomes and two MoS outcomes—were treated as a single primary family of tests. The false discovery rate (FDR) was controlled using the Benjamini–Hochberg procedure (Benjamini and Hochberg, 1995). All tests were two-tailed, and an FDR-adjusted q < 0.05 served as the primary criterion for statistical significance. Both uncorrected p values and FDR-adjusted q values are reported, with the q value serving as the primary decision criterion. For outcomes with a significant omnibus test, pairwise comparisons between slope conditions were performed using the emmeans package (Lenth, 2023). For each outcome, the family-wise error rate across the 10 pairwise comparisons was controlled using the Holm procedure (Holm, 1979). *Post hoc* comparisons are reported as the estimated mean difference, its 95% confidence interval (CI), the Holm-adjusted p value, and the paired standardized effect size dz. When the omnibus q value was ≥ 0.05, individual Holm-adjusted comparisons reaching p < 0.05 were not interpreted further. Model diagnostics included checks for singular fits, convergence problems, residual normality using the Shapiro–Wilk test and quantile–quantile plots, and standardized residuals.

The mean ± SD of the raw peak values for the left and right limbs at each slope, together with the proportion of participants showing R > L, are presented in **Table 1**. Descriptively, higher right-limb values were more frequent for peak ankle plantarflexion moment and peak Achilles tendon strain, whereas higher left-limb values were more frequent for peak knee extension moment and peak patellofemoral stress. The direction of peak vGRF was more evenly distributed across participants. These proportions describe anatomical left–right differences and were interpreted only as descriptive indicators of asymmetry direction for each outcome.

**Table 1:** Descriptive statistics of raw right- and left-limb peak values (mean ± SD) and asymmetry direction across slopes.

Outcome	Limb/Direction	−6°	−3°	0°	+3°	+6°	Unit
Peak vGRF	Right	2.474 ± 0.28	2.426 ± 0.23	2.426 ± 0.18	2.398 ± 0.19	2.353 ± 0.18	BW
	Left	2.459 ± 0.32	2.432 ± 0.27	2.419 ± 0.21	2.386 ± 0.21	2.349 ± 0.20	BW
	R>L %	68.4%	52.6%	57.9%	63.2%	57.9%	
Peak ankle PF moment	Right	1.854 ± 0.39	2.401 ± 0.45	2.953 ± 0.48	3.613 ± 0.48	4.211 ± 0.62	N·m·kg⁻¹
	Left	1.736 ± 0.41	2.189 ± 0.49	2.746 ± 0.54	3.396 ± 0.53	3.995 ± 0.68	N·m·kg⁻¹
	R>L %	63.2%	100%	89.5%	89.5%	84.2%	
Peak Achilles tendon strain	Right	2.762 ± 0.48	3.747 ± 0.52	4.922 ± 0.79	6.350 ± 0.85	7.714 ± 1.06	%
	Left	2.523 ± 0.59	3.467 ± 0.61	4.643 ± 0.89	6.066 ± 0.98	7.371 ± 1.14	%
	R>L %	89.5%	84.2%	84.2%	73.7%	78.9%	
Peak knee ext moment	Right	3.968 ± 0.51	3.079 ± 0.39	2.243 ± 0.44	1.450 ± 0.34	1.113 ± 0.25	N·m·kg⁻¹
	Left	4.030 ± 0.49	3.187 ± 0.47	2.363 ± 0.44	1.572 ± 0.33	1.093 ± 0.23	N·m·kg⁻¹
	R>L %	42.1%	31.6%	47.4%	31.6%	52.6%	
Peak PFJ stress	Right	10.993 ± 1.34	8.977 ± 1.49	7.529 ± 1.27	6.369 ± 1.00	5.458 ± 1.05	MPa
	Left	11.145 ± 1.33	9.322 ± 1.56	7.907 ± 1.24	6.771 ± 1.03	5.808 ± 1.08	MPa
	R>L %	42.1%	31.6%	31.6%	26.3%	26.3%	

The “Right” and “Left” rows show the group mean ± SD of the right- (R) and left-limb (L) peak values at each slope; the “R>L %” row shows the proportion of participants whose right-limb peak exceeded the left; n = 19. Side-specific values and direction are reported descriptively only; no per-slope inferential test of limb side was performed. vGRF, vertical ground reaction force; PF, plantarflexion; ext, extension; PFJ, patellofemoral joint; BW, body weight.

**Figure 1:**
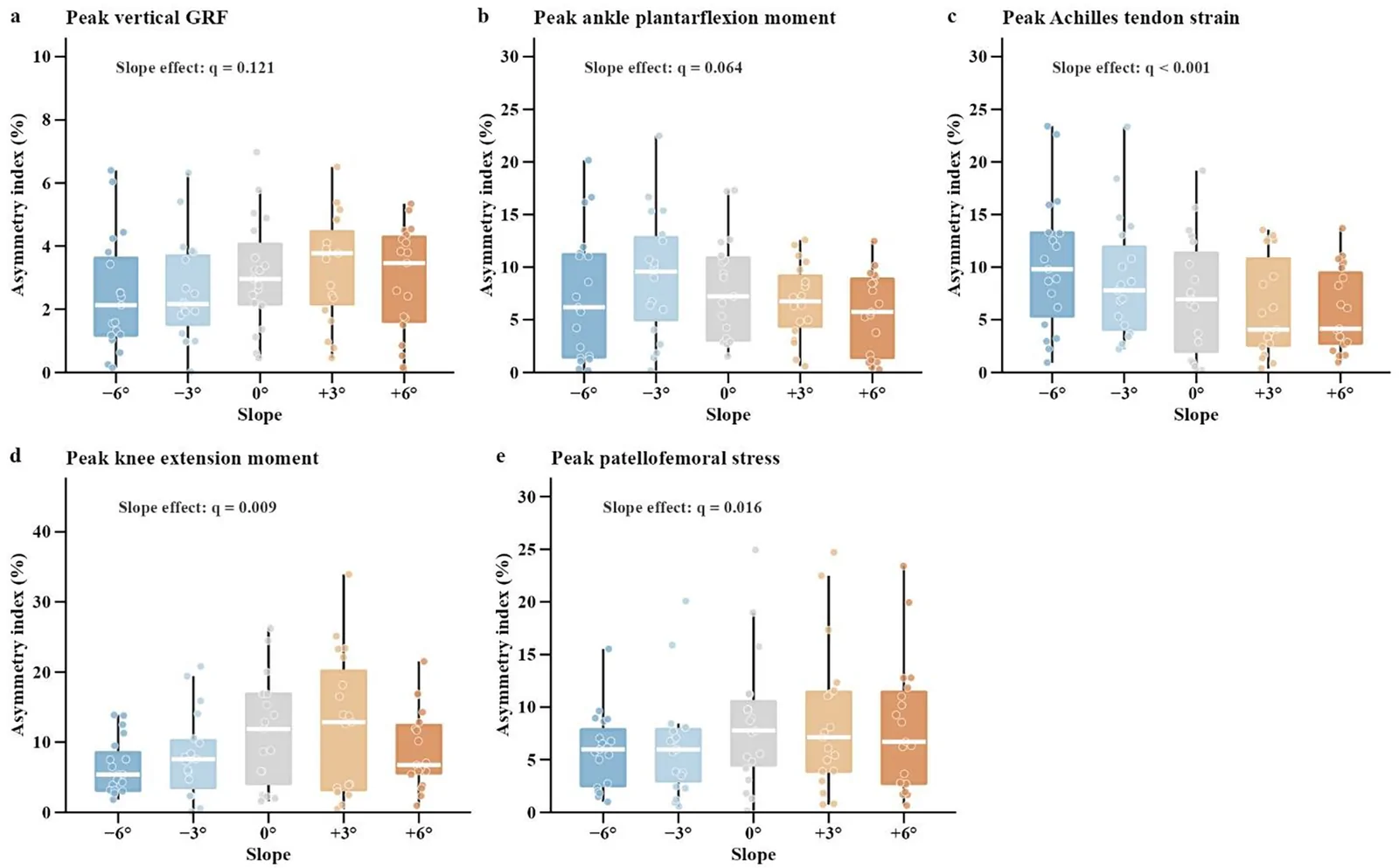
Asymmetry indices for the five peak outcomes across running slopes. Panels show the asymmetry index (%) at each slope (−6°, −3°, 0°, +3°, and +6°) for **(a)** peak vertical ground reaction force, **(b)** peak ankle plantarflexion moment, **(c)** peak Achilles tendon strain, **(d)** peak knee extension moment, and **(e)** peak patellofemoral stress. The Asymmetry Index was calculated using the standard percentage difference method. In each box plot, the central white line denotes the median, the box represents the interquartile range, and the whiskers extend to 1.5 times the interquartile range; circles represent individual participants (n = 19). The q value shown in each panel is the Benjamini–Hochberg false-discovery-rate-adjusted p value for the omnibus slope effect from the linear mixed-effects model, with adjustment applied across the seven primary outcomes.

After Benjamini–Hochberg FDR correction, a significant slope effect was found for the asymmetry index in peak Achilles tendon strain (F_4_, _72_ = 6.09, p < 0.001, q < 0.001, partial η² = 0.25), which decreased monotonically from downhill to uphill slopes (**Table 2**; **Figure 1c**). *Post hoc* comparisons showed that the −6°condition had a significantly greater percentage difference than the 0°condition (mean difference = 3.03 percentage points, 95% CI: 0.94 to 5.12, dz = 0.66, Holm-adjusted p = 0.041), the +3°condition (mean difference = 4.07 percentage points, 95% CI: 1.98 to 6.17, dz = 0.89, Holm-adjusted p = 0.002), and the +6°condition (mean difference = 4.34 percentage points, 95% CI: 2.25 to 6.43, dz = 0.95, Holm-adjusted p < 0.001). No other pairwise comparison between slopes reached significance. A significant slope effect was also found for the asymmetry index in peak knee extension moment (F_4_, _72_ = 4.21, p = 0.004, q = 0.009, partial η² = 0.19), which peaked at +3° (**Table 2**; **Figure 1d**). *Post hoc* comparisons showed that the percentage difference was significantly greater in the 0°condition than in the −6°condition (mean difference = 4.85 percentage points, 95% CI: 1.51 to 8.18, dz = 0.66, Holm-adjusted p = 0.045) and significantly greater in the +3°condition than in the −6°condition (mean difference = 6.01 percentage points, 95% CI: 2.67 to 9.35, dz = 0.82, Holm-adjusted p = 0.006). No other pairwise comparison between slopes reached significance. The asymmetry index for peak patellofemoral stress also differed significantly across slopes (F_4_, _72_ = 3.62, p = 0.010, q = 0.016, partial η² = 0.17; **Table 2**; **Figure 1e**); however, none of the Holm-adjusted pairwise comparisons reached significance (all adjusted p ≥ 0.055). The asymmetry indices for peak vGRF and peak ankle plantarflexion moment did not meet the primary criterion for significance (q = 0.121 and q = 0.064, respectively; partial η² ≤ 0.12; **Figures 1a, b**), providing no statistically significant evidence that the magnitude of their bilateral differences varied across slopes at a fixed running speed.

**Table 2:** Asymmetry index (mean ± SD) and main effect of slope.

Outcome	−6°	−3°	0°	+3°	+6°	*F*_(4, 72)_	*q*	Partial η^2^
Peak vGRF	2.46 ± 1.83	2.54 ± 1.60	3.12 ± 1.74	3.32 ± 1.68	2.89 ± 1.73	1.89	0.121	0.10
Peak ankle PF moment	7.28 ± 6.14	9.17 ± 5.91	7.87 ± 4.90	6.73 ± 3.53	5.65 ± 3.86	2.44	0.064	0.12
Peak Achilles tendon strain	10.25 ± 6.39	8.79 ± 5.79	7.22 ± 5.65	6.18 ± 4.69	5.91 ± 3.94	6.09	<0.001	0.25
Peak knee ext moment	6.45 ± 3.95	8.08 ± 5.95	11.30 ± 7.78	12.46 ± 9.96	8.96 ± 5.58	4.21	0.009	0.19
Peak PFJ stress	5.88 ± 3.61	6.27 ± 4.92	8.47 ± 6.23	8.64 ± 6.84	8.11 ± 6.27	3.62	0.016	0.17

Values are the asymmetry indices (mean ± SD) expressed in percentage points; n = 19. F represents the omnibus test of slope from the linear mixed-effects model (denominator degrees of freedom = 72), and q represents the Benjamini–Hochberg false-discovery-rate-adjusted p value across the seven primary outcomes. Significant pairwise comparisons between slopes (mean difference, 95% confidence interval, paired standardized effect size dz, and Holm-adjusted p value) are reported in the text. SD, standard deviation; vGRF, vertical ground reaction force; PF, plantarflexion; ext, extension; PFJ, patellofemoral joint.

**Figure 2:**
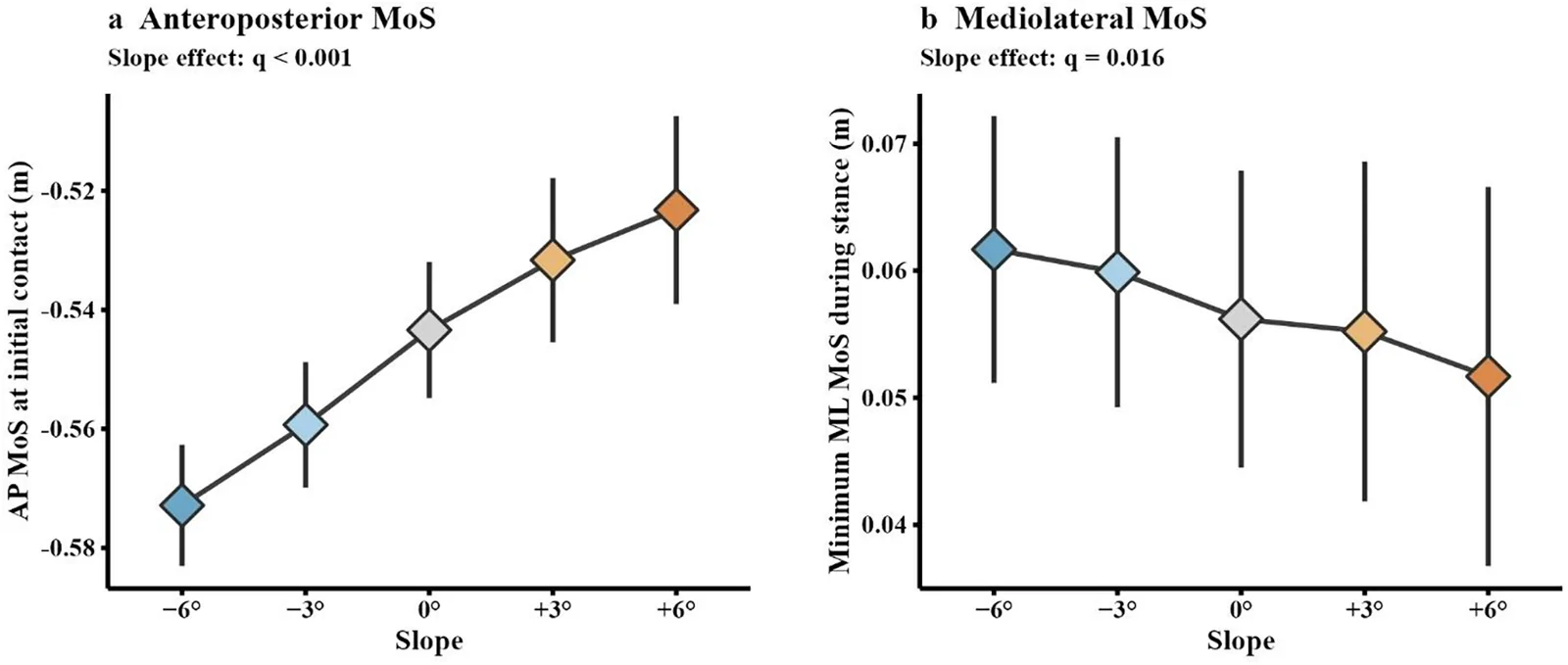
Dynamic stability across running slopes. **(a)** Anteroposterior (AP) margin of stability (MoS) at initial contact and **(b)** minimum mediolateral (ML) MoS at each slope (−6°, −3°, 0°, +3°, +6°). Symbols denote group means and error bars the 95% confidence intervals (n = 19); connecting lines indicate slope order only. The q value in each panel is the Benjamini–Hochberg false-discovery-rate-adjusted p value for the omnibus slope effect from the linear mixed-effects model across the seven primary outcomes.

After correcting AP velocity for treadmill belt motion, a significant slope effect was observed for AP MoS (F_4_, _72_ = 77.74, p < 0.001, q < 0.001, partial η² = 0.81; **Table 3**; **Figure 2a**). AP MoS increased progressively, becoming less negative from −0.573 ± 0.021 m at −6° to −0.523 ± 0.033 m at +6°. All 10 Holm-adjusted pairwise comparisons were significant (adjusted p ≤ 0.011), with absolute mean differences ranging from 0.0084 m between +3° and +6° (95% CI, 0.0020 to 0.0149; dz = 0.91) to 0.0496 m between −6° and +6° (95% CI, 0.0432 to 0.0561; dz = 2.36). A significant slope effect was also observed for ML MoS (F_4_, _72_ = 3.50, p = 0.011, q = 0.016, partial η² = 0.16; **Table 3**; **Figure 2b**), which decreased from 0.062 ± 0.022 m at −6° to 0.052 ± 0.031 m at +6°. ML MoS was significantly greater at −6° than at +6°, with a mean difference of 0.0100 m (95% CI, 0.0041 to 0.0159; dz = 0.52; Holm-adjusted p = 0.013), whereas no other pairwise comparison was significant (adjusted p ≥ 0.067).

**Table 3:** Dynamic stability (mean ± SD) and main effect of slope.

Outcome	−6°	−3°	0°	+3°	+6°	F_(4, 72)_	q	Partial η^2^
AP MoS at initial contact	−0.573 ± 0.02	−0.559 ± 0.02	−0.543 ± 0.02	−0.532 ± 0.03	−0.523 ± 0.03	77.74	<0.001	0.81
Minimum ML MoS	0.062 ± 0.02	0.060 ± 0.02	0.056 ± 0.02	0.055 ± 0.03	0.052 ± 0.03	3.5	0.016	0.16

Values are the mean ± SD of participant condition means, in meters. F represents the omnibus test of slope from the linear mixed-effects model (denominator degrees of freedom = 72), and q represents the Benjamini–Hochberg false-discovery-rate-adjusted p value across the seven primary outcomes. Significant pairwise comparisons are reported in the text. AP, anteroposterior; ML, mediolateral; MoS, margin of stability; SD, standard deviation.

## 3 Discussion

This study examined differences in lower-limb bilateral asymmetry and dynamic stability across slopes at a fixed speed and yielded three main findings. First, site-specific differences in bilateral asymmetry and dynamic stability were observed across slopes. Significant slope effects were observed for the asymmetry of Achilles tendon strain, knee extension moment, and patellofemoral stress, as well as for both AP and ML MoS, whereas no significant slope effects were found for vertical ground reaction force or ankle plantarflexion moment asymmetry. The direction of asymmetry likewise differed by site, with the ankle and Achilles tendon more often larger on the right, the knee and patellofemoral joint more often larger on the left, and the vertical external force more evenly distributed. This inconsistency in direction across outcomes and participants suggests that asymmetry reflects individualized, outcome-specific loading patterns and supports considering asymmetry magnitude separately from its direction. Second, load magnitude and bilateral asymmetry did not change in parallel, and in some cases changed in opposite directions. Achilles tendon strain increased from downhill to uphill slopes, a loading trend consistent with previous work, yet its bilateral asymmetry decreased progressively with uphill slope; that is, the greater the load, the more even the distribution between limbs. This inverse relationship is a novel observation of the present study. The loading trend for knee extension moment was the reverse, decreasing progressively with uphill slope and being highest downhill, whereas its asymmetry was greatest at mild uphill. Load magnitude and bilateral asymmetry therefore did not change in parallel at either site, indicating that unilateral peak magnitude alone may not fully characterize bilateral load distribution. Third, after AP velocity was expressed relative to treadmill-belt motion, AP MoS was strongly negative but increased monotonically, becoming less negative from downhill to uphill running. ML MoS decreased more modestly across slopes, with a significant difference between −6° and +6°. These MoS findings describe direction- and event-specific instantaneous mechanical margins and should not be interpreted as direct measures of global locomotor stability.

There is ample evidence that slope alters the distribution of load within the lower limb. DeVita et al. (2008) found that, during non-level running, joint work in the lower limb was biased from energy dissipation toward energy generation and was redistributed among the ankle, knee, and hip with slope; during uphill running the lower-limb muscles perform more net mechanical work to increase the potential energy of the body, whereas downhill running is dominated by energy dissipation (Vernillo et al., 2017). In the present study, the ankle plantarflexion moment increased and the knee extension moment decreased with uphill slope, consistent with this pattern of load redistribution. Previous work, however, has largely addressed the magnitude of load with both limbs combined. The present study further shows that differences across slopes were site-specific among the seven primary outcomes. Significant effects were observed for the asymmetry of Achilles tendon strain, knee extension moment, and patellofemoral stress, as well as for both AP and ML MoS, whereas no significant effects were found for vertical ground reaction force or ankle plantarflexion moment asymmetry. This site-specific pattern may be related to the predominantly sagittal-plane effects of slope on lower-limb loading (DeVita et al., 2008; Vernillo et al., 2017), although the present analysis cannot establish the underlying mechanism. Together with the inconsistent direction of asymmetry across participants, these findings suggest a local, structure-specific redistribution of load rather than a uniform bilateral response.

Van Hooren et al. (2024) reported that steeper uphill slopes increase Achilles tendon force and strain, and thus its cumulative load, owing to a greater prevalence of forefoot landing, whereas downhill running reduces it comparatively. The present study confirmed this trend of increasing Achilles tendon load with uphill slope, but further found that its bilateral asymmetry decreased rather than increased with uphill slope; that is, the greater the load, the more evenly it was distributed between limbs. This inverse relationship has not been reported previously. The knee extension moment showed the opposite pattern, with load highest downhill but asymmetry greatest at mild uphill. As to the underlying mechanism, one possible explanation is that high-load conditions compel both limbs to be recruited more fully, thereby compressing left–right differences, whereas lower loads leave more scope for individualized left–right loading patterns. This explanation is consistent with evidence that the effects of load and fatigue on asymmetry vary across tasks and metrics. For example, during running, vertical stiffness and loading rate have been reported to become more symmetrical after fatigue (Radzak et al., 2017), while higher running speeds are often accompanied by smaller ground reaction force asymmetry (Mo et al., 2020). Similarly, D’Hondt et al. (2026a) found that a fatigue-inducing protocol increased asymmetry only in the repeated single-leg 10-s jump, but not in single-leg countermovement or horizontal jump tests, supporting the task-specific nature of fatigue-related asymmetry responses. Other variables, however, show the opposite trend; therefore, the relationship between load and symmetry remains inconsistent, and the mechanism proposed here requires direct verification. Taken together, these divergent patterns suggest that bilateral asymmetry may provide information complementary to load magnitude. However, their relationship was not formally tested, and this interpretation requires confirmation in larger studies.

Bilateral asymmetry has been regarded as a functionally meaningful but task- and outcome-specific construct across sporting tasks. In speed climbing, lower-limb asymmetry has been related to performance and appears to be driven primarily by neural activation rather than differences in muscle thickness (Zhang et al., 2025). This interpretation is supported by evidence that lower-limb performance in speed climbers depends more on neuromuscular activation than on muscle morphology alone (Li et al., 2026). Comparable sport-specific findings have been reported elsewhere: jumping asymmetries were associated with poorer sprint, change-of-direction, and jump performance in elite academy soccer players (Bishop et al., 2021), whereas morphological asymmetry in youth judokas was generally small and unrelated to competitive level or sex (D’Hondt et al., 2025b). The present findings indicate that asymmetry direction varied across individuals and outcomes, supporting the interpretation that bilateral asymmetry reflects outcome-specific and individualized neuromuscular control. Building on this, the present study further observed that the slopes associated with the highest mean asymmetry values did not coincide with those associated with the highest absolute loads. Mean Achilles tendon asymmetry was highest during downhill running whereas its absolute load was highest uphill, while the mean asymmetry indices for knee extension moment and patellofemoral stress were highest at the mild uphill slope (+3°), whereas their absolute loads were highest downhill. However, for patellofemoral stress asymmetry, none of the Holm-adjusted pairwise comparisons between slopes reached significance. Thus, identifying the condition with the greatest potential for uneven loading requires consideration of both load magnitude and bilateral distribution rather than absolute load alone. Moreover, because asymmetry direction varied across individuals, the limb bearing the higher load should be identified individually rather than attributed to a fixed side. The relationship between asymmetry and injury remains uncertain. The cautious interpretation of injury implications adopted here is in line with Malisoux et al. (2024), who reported that asymmetry in spatiotemporal and kinetic variables measured during treadmill running at a preferred speed was not associated with a higher risk of running-related injury. The present findings provide additional context by showing that asymmetry varies across slopes for selected joint- and tissue-loading variables but not for others. This indicates that running asymmetry is condition- and outcome-specific; consequently, asymmetry measured using spatiotemporal or ground-reaction-force variables under a single running condition may not fully represent bilateral joint and tissue loading across slopes. Nevertheless, whether these slope-related asymmetry patterns are associated with injury risk remains to be examined prospectively.

The corrected AP results reflect the requirement that treadmill locomotion be expressed relative to the moving support surface: treadmill-belt velocity must be incorporated into laboratory-frame AP velocity before calculating the XCoM (Curtze et al., 2024). This transformation shifted the AP XCoM anteriorly and produced AP MoS values below −0.50 m. Such negative values are mechanically plausible during running and should not be interpreted as evidence of global instability. The monotonic increase from −6° to +6° indicates that the signed AP margin became progressively less negative during uphill running. In the present convention, a less negative AP MoS represents a larger instantaneous AP mechanical margin; however, this does not establish that uphill running was globally more stable, because the observed change may also reflect a compensatory adjustment in gait organization. ML MoS decreased more modestly across slopes, with the clearest difference occurring between −6° and +6°. These results can be considered alongside those of Promsri (2025), who analyzed the same dataset using the largest Lyapunov exponent and reported lower local dynamic stability during downhill than uphill running, particularly during mid-stance. The largest Lyapunov exponent reflects sensitivity of movement dynamics to small perturbations, whereas MoS describes the instantaneous mechanical relationship between the XCoM and the base of support; therefore, the two measures are complementary but not directly interchangeable. Evidence from walking suggests that ML stability depends mainly on step-by-step foot placement, whereas AP stability may additionally involve modulation of ankle push-off (Bruijn and Van Dieën, 2018). However, whether ankle push-off serves a comparable stabilizing function during running remains unclear, limiting the transferability of this walking-based explanation. Accordingly, the present data demonstrate slope-related differences in AP and ML MoS but cannot determine the underlying control mechanisms. Because ground-contact time and step width were not examined, it remains unclear whether these patterns primarily reflect changes in gait organization, balance-control mechanisms, or both. Their implications for perturbation recovery and injury risk also remain speculative because neither perturbation responses nor injuries were assessed.

This study has several limitations. First, the analysis was conducted at a single running speed (2.78 m·s⁻¹), and the sample ranged from untrained runners to Olympic-level athletes without training level being included as a moderator. These factors limit generalization to other running speeds and specific performance levels, while differences in training experience and habitual technique may have contributed to the between-participant variability in asymmetry. Second, pre-existing lower-limb anatomical asymmetries, such as leg-length discrepancy and knee Q-angle, were unavailable in the public dataset and could not be controlled. These factors may have contributed to baseline left–right differences or modified individual responses to slope; therefore, the observed asymmetry patterns cannot be attributed solely to slope-induced neuromuscular adjustments. Third, despite the sensitivity analysis, the modest sample size may have limited the detection of smaller slope effects. Accordingly, the nonsignificant findings, particularly for vertical ground reaction force and ankle plantarflexion moment asymmetry, should not be interpreted as evidence of no slope effect. Fourth, dynamic stability was estimated using the geometric center of four pelvic markers as a proxy for the whole-body center of mass rather than a full-body segmental calculation. This approximation may introduce systematic deviation in absolute MoS values; therefore, the reported MoS values should be interpreted as estimates rather than direct whole-body measurements. Fifth, the AP transformation assumed a constant treadmill-belt speed of 2.78 m·s⁻¹ and represented the treadmill surface using the nominal slope; information on small deviations in actual belt speed or treadmill-plane calibration was unavailable. Sixth, asymmetry direction was assessed descriptively using anatomical left–right comparisons without inferential testing of limb side. These directional findings should therefore be regarded as exploratory and outcome-specific. Future studies should incorporate multiple running speeds, anatomical assessments, larger and training-stratified samples, full-body segmental center-of-mass calculations, and formal slope × side analyses.

## 4 Conclusion

At a fixed running speed, lower-limb bilateral load distribution and direction-specific mechanical margins varied across slopes. Differences across slopes were observed in the asymmetry of Achilles tendon strain, knee extension moment, and patellofemoral stress and in both AP and ML MoS, whereas no statistically significant differences were detected for vertical ground reaction force or ankle plantarflexion moment asymmetry. After AP velocity was expressed relative to treadmill-belt motion, AP MoS was strongly negative and became progressively less negative from downhill to uphill running, whereas ML MoS decreased more modestly. Asymmetry magnitude did not vary in parallel with absolute load, supporting the consideration of bilateral load distribution alongside absolute loading. However, the corrected MoS values represent direction- and event-specific instantaneous mechanical margins rather than direct measures of global locomotor stability. Because training adaptations, perturbation responses, performance, and injury outcomes were not assessed, the practical implications remain preliminary.

## Data Availability

Publicly available datasets were analyzed in this study. This data can be found here: https://osf.io/7qbxc.

## References

[B1] Afonso J. Virgile A. Peña J. Jordan M. García-de-Alcaraz A. Sá M. . (2025). On uneven ground: embracing the challenges of inter-limb asymmetries and their assessment. Asymmetry 2, 4. doi: 10.55092/asymmetry20250004

[B2] Alenezi F. Herrington L. Jones P. Jones R. (2016). How reliable are lower limb biomechanical variables during running and cutting tasks? J. Electromyogr. Kinesiol. 30, 137–142. doi: 10.1016/j.jelekin.2016.07.001 27403855

[B3] Arellano C. J. Kram R. (2012). The energetic cost of maintaining lateral balance during human running. J. Appl. Physiol. 112, 427–434. doi: 10.1152/japplphysiol.00554.2011 22052870

[B4] Bates D. Mächler M. Bolker B. Walker S. (2015). Fitting linear mixed-effects models using lme4. J. Stat. Software 67, 1–48. doi: 10.18637/jss.v067.i01

[B5] Benjamini Y. Hochberg Y. (1995). Controlling the false discovery rate: a practical and powerful approach to multiple testing. J. R. Stat. Soc Ser. B. Stat. Methodol. 57, 289–300. doi: 10.1111/j.2517-6161.1995.tb02031.x40046247

[B6] Bertelsen M. L. Hulme A. Petersen J. Brund R. K. Sørensen H. Finch C. F. . (2017). A framework for the etiology of running-related injuries. Scand. J. Med. Sci. Sports 27, 1170–1180. doi: 10.1111/sms.12883 28329441

[B7] Bishop C. Read P. Lake J. Chavda S. Turner A. (2018). Interlimb asymmetries: understanding how to calculate differences from bilateral and unilateral tests. Strength Cond. J. 40, 1–6. doi: 10.1519/SSC.000000000000037138604988

[B8] Bishop C. Brashill C. Abbott W. Read P. Lake J. Turner A. (2021). Jumping asymmetries are associated with speed, change of direction speed, and jump performance in elite academy soccer players. J. Strength Cond. Res. 35, 1841–1847. doi: 10.1519/JSC.0000000000003058 30707141

[B9] Bishop C. de Keijzer K. L. Turner A. N. Beato M. (2023). Measuring interlimb asymmetry for strength and power: a brief review of assessment methods, data analysis, current evidence, and practical recommendations. J. Strength Cond. Res. 37, 745–750. doi: 10.1519/JSC.0000000000004384 36695841

[B10] Bruijn S. M. Van Dieën J. H. (2018). Control of human gait stability through foot placement. J. R. Soc Interface 15, 20170816. doi: 10.1098/rsif.2017.0816 29875279 PMC6030625

[B11] Catelli D. S. Wesseling M. Jonkers I. Lamontagne M. (2019). A musculoskeletal model customized for squatting task. Comput. Methods Biomech. Biomed. Engin. 22, 21–24. doi: 10.1080/10255842.2018.1523396 30398067

[B12] Chapelle L. Bishop C. D’Hondt J. D’Hondt E. Clarys P. (2022). Morphological and functional asymmetry in elite youth tennis players compared to sex- and age-matched controls. J. Sports Sci. 40, 1618–1628. doi: 10.1080/02640414.2022.2096769 35830516

[B13] Curtze C. Buurke T. J. W. McCrum C. (2024). Notes on the margin of stability. J. Biomech. 166, 112045. doi: 10.1016/j.jbiomech.2024.112045 38484652

[B14] D’Hondt J. Bishop C. Chapelle L. De Pauw K. Clarys P. D’Hondt E. . (2025a). Consistency between lower limb preference and dominance in healthy adult distance runners: exploring its variability across tasks and time. Res. Square. doi: 10.21203/rs.3.rs-8147618/v142669180. Preprint.

[B15] D’Hondt J. Chapelle L. Bishop C. Aerenhouts D. De Pauw K. Clarys P. . (2024). Association between inter-limb asymmetry and determinants of middle- and long-distance running performance in healthy populations: a systematic review. Sports Med. - Open 10, 127. doi: 10.1186/s40798-024-00790-w 39589611 PMC11599690

[B16] D’Hondt J. Chapelle L. Lindekens T. Clarys P. (2025b). Morphological inter-limb asymmetry in youth judokas is independent of competitive level and sex. BMC Sports Sci. Med. Rehabil. 17, 380. doi: 10.1186/s13102-025-01440-8 41286933 PMC12751752

[B17] D’Hondt J. Denayer R. Verstraeten S. Clarys P. Chapelle L. (2026b). In-water 505 deficit as an isolated measure of directional performance and asymmetry in male water polo athletes. Int. J. Sports Physiol. Perform., 1–7. doi: 10.1123/ijspp.2025-0677 42501976

[B18] D’Hondt J. Vanhoudt M. Dani M. Chapelle L. Aerenhouts D. (2026a). Does a muscle fatigue-inducing protocol alter the magnitude of jump inter-limb asymmetry in healthy adolescents? J. Hum. Kinet. 103, 57–68. doi: 10.5114/jhk/205705 PMC13551332 42712858

[B19] De Groote F. Kinney A. L. Rao A. V. Fregly B. J. (2016). Evaluation of direct collocation optimal control problem formulations for solving the muscle redundancy problem. Ann. Biomed. Eng. 44, 2922–2936. doi: 10.1007/s10439-016-1591-9 27001399 PMC5043004

[B20] Delp S. L. Anderson F. C. Arnold A. S. Loan P. Habib A. John C. T. . (2007). OpenSim: open-source software to create and analyze dynamic simulations of movement. IEEE Trans. Biomed. Eng. 54, 1940–1950. doi: 10.1109/TBME.2007.901024 18018689

[B21] DeVita P. Janshen L. Rider P. Solnik S. Hortobágyi T. (2008). Muscle work is biased toward energy generation over dissipation in non-level running. J. Biomech. 41, 3354–3359. doi: 10.1016/j.jbiomech.2008.09.024 19010471 PMC2590776

[B22] Drama Ö. Vielemeyer J. Badri-Spröwitz A. Müller R. (2020). Postural stability in human running with step-down perturbations: an experimental and numerical study. R. Soc Open Sci. 7, 200570. doi: 10.1098/rsos.200570 33391782 PMC7735328

[B23] Hof A. L. Gazendam M. G. J. Sinke W. E. (2005). The condition for dynamic stability. J. Biomech. 38, 1–8. doi: 10.1016/j.jbiomech.2004.03.025 15519333

[B24] Holm S. (1979). A simple sequentially rejective multiple test procedure. Scand. J. Stat. 6, 65–70.

[B25] Kakouris N. Yener N. Fong D. T. P. (2021). A systematic review of running-related musculoskeletal injuries in runners. J. Sport Health Sci. 10, 513–522. doi: 10.1016/j.jshs.2021.04.001 33862272 PMC8500811

[B26] Kuznetsova A. Brockhoff P. B. Christensen R. H. B. (2017). lmerTest package: tests in linear mixed effects models. J. Stat. Software 82, 1–26. doi: 10.18637/jss.v082.i13

[B27] Lenth R. V. (2023). emmeans: estimated marginal means, aka least-squares means R package version 1.8.9. Available online at: https://CRAN.R-project.org/package=emmeans (Accessed September 3, 2026).

[B28] Li M. Zhu Y. Peng Y. (2026). The relationship between lower-limb explosive strength and climbing speed in elite speed climbers: a comprehensive analysis based on muscle morphology and electromyography. Int. J. Sports Physiol. Perform. 21, 456–462. doi: 10.1123/ijspp.2025-0329 41666905

[B29] Malisoux L. Gette P. Delattre N. Urhausen A. Theisen D. (2024). Gait asymmetry in spatiotemporal and kinetic variables does not increase running-related injury risk in lower limbs: a secondary analysis of a randomised trial including 800+ recreational runners. BMJ Open Sport Exerc. Med. 10, e001787. doi: 10.1136/bmjsem-2023-001787 38196940 PMC10773390

[B30] Mo S. Lau F. O. Y. Lok A. K. Y. Chan Z. Y. S. Zhang J. H. Shum G. . (2020). Bilateral asymmetry of running gait in competitive, recreational and novice runners at different speeds. Hum. Mov. Sci. 71, 102600. doi: 10.1016/j.humov.2020.102600 32174449

[B31] Nüesch C. Overberg J.-A. Schwameder H. Pagenstert G. Mündermann A. (2018). Repeatability of spatiotemporal, plantar pressure and force parameters during treadmill walking and running. Gait Posture 62, 117–123. doi: 10.1016/j.gaitpost.2018.03.017 29547791

[B32] Park S.-K. Jeon H.-M. Lam W.-K. Stefanyshyn D. Ryu J. (2019). The effects of downhill slope on kinematics and kinetics of the lower extremity joints during running. Gait Posture 68, 181–186. doi: 10.1016/j.gaitpost.2018.11.007 30497038

[B33] Promsri A. (2025). Neuromuscular control in incline and decline treadmill running: insights into movement synergies for training and rehabilitation. Signals 6, 2. doi: 10.3390/signals601000230654563

[B34] Radzak K. N. Putnam A. M. Tamura K. Hetzler R. K. Stickley C. D. (2017). Asymmetry between lower limbs during rested and fatigued state running gait in healthy individuals. Gait Posture 51, 268–274. doi: 10.1016/j.gaitpost.2016.11.005 27842295

[B35] Silverman A. K. Wilken J. M. Sinitski E. H. Neptune R. R. (2012). Whole-body angular momentum in incline and decline walking. J. Biomech. 45, 965–971. doi: 10.1016/j.jbiomech.2012.01.012 22325978

[B36] Steele K. M. DeMers M. S. Schwartz M. H. Delp S. L. (2012). Compressive tibiofemoral force during crouch gait. Gait Posture 35, 556–560. doi: 10.1016/j.gaitpost.2011.11.023 22206783 PMC3319529

[B37] van den Bogert A. J. Geijtenbeek T. Even-Zohar O. Steenbrink F. Hardin E. C. (2013). A real-time system for biomechanical analysis of human movement and muscle function. Med. Biol. Eng. Comput. 51, 1069–1077. doi: 10.1007/s11517-013-1076-z 23884905 PMC3751375

[B38] Van Hooren B. Meijer K. (2024). Dataset of running kinematics, kinetics and muscle activation at different speeds, surface gradients, cadences and with forward trunk lean. Data Brief 54, 110312. doi: 10.1016/j.dib.2024.110312 39822723 PMC11737525

[B39] Van Hooren B. van Rengs L. Meijer K. (2023). “ Dataset of running biomechanics across different speeds, gradients and cadences,” in Open Science Framework. Dataset. (Charlottesville, VA: Center for Open Science). doi: 10.17605/OSF.IO/7QBXC

[B40] Van Hooren B. van Rengs L. Meijer K. (2024). Per-step and cumulative load at three common running injury locations: the effect of speed, surface gradient, and cadence. Scand. J. Med. Sci. Sports 34, e14570. doi: 10.1111/sms.14570 38389144

[B41] Vernillo G. Giandolini M. Edwards W. B. Morin J.-B. Samozino P. Horvais N. . (2017). Biomechanics and physiology of uphill and downhill running. Sports Med. 47, 615–629. doi: 10.1007/s40279-016-0605-y 27501719

[B42] Viljoen C. Janse van Rensburg D. C. van Mechelen W. Verhagen E. Silva B. Scheer V. . (2022). Trail running injury risk factors: a living systematic review. Br. J. Sports Med. 56, 577–587. doi: 10.1136/bjsports-2021-104858 35022162

[B43] Woltring H. J. (1986). A Fortran package for generalized, cross-validatory spline smoothing and differentiation. Adv. Eng. Software 8, 104–113. doi: 10.1016/0141-1195(86)90098-7

[B44] Zhang X. Zhu C. Ou Z. Wu L. Li M. (2025). The relationship between lower-limb asymmetry and speed-climbing time: the role of muscle thickness and neural activation. Int. J. Sports Physiol. Perform. 20, 1161–1167. doi: 10.1123/ijspp.2025-0052 40581348

